# Defensive tolerance to parasitism is correlated with sexual selection in swallows

**DOI:** 10.1007/s00442-023-05419-5

**Published:** 2023-07-18

**Authors:** Juan José Soler, Anders Pape Møller

**Affiliations:** 1 Depto. Ecología Funcional Y Evolutiva, Estación Experimental de Zonas Áridas, Sacramento S/N, La Cañada de San Urbano, 04120 Almería, Spain; 2 Ecologie Systématique Evolution, Université Paris-Sud, CNRS, Orsay, France

**Keywords:** Host-parasite interactions, Mate choice, Sexual selection, Tolerance to parasitism

## Abstract

Parasite-mediated sexual selection has been the topic of extensive research and enthusiastic debate for more than three decades. Here, we suggest that secondary sexual characters may not only signal parasite resistance but also defensive tolerance. We exemplify this possibility by analysing information on two sexually selected traits, annual reproductive success, and ectoparasitism in a barn swallow *Hirundo rustica* population followed for more than 30 years. For each individual, we estimated the slope of the association between reproductive success and parasitism as an index of tolerance and subsequently explored the association with the expression of the sexually selected traits. In accordance with expectations of parasites playing a role in sexual selection, tail length was negatively related to load of chewing lice and nest size was positively related to tolerance to chewing lice. We discuss the importance of considering defensive tolerance for understanding the role of parasite-mediated sexual selection.

**Supplementary Information:**

The online version contains supplementary material available at 10.1007/s00442-023-05419-5.

## Introduction

The study of the role of parasitism and immunity in sexual selection has been one of the most fruitful areas of research during the last decades (Schmid-Hempel 2011). The general hypothesis posits that individuals by choosing mates with low parasite loads would experience direct and indirect fitness advantages (Andersson 1994). By selecting partners with characteristics reflecting reduced parasite loads, the choosy sex may benefit directly from a reduced risk of parasite infections (i.e. transmission avoidance model), or from acquiring better quality territories in terms of reduced risk of parasitism or resource availability for use in antiparasitic defence (i.e. resource provisioning model) (Clayton 1991). In addition, if resistance to parasitism has a genetic component, individuals choosing a partner with traits reflecting such resistance would achieve indirect genetic benefits by acquiring these good genes for their offspring (indicator models of resistance to parasitism (Mead and Arnold 2004)). Thus, scenarios of parasite-mediated sexual selection would directly influence the strength of selection due to parasitism, but also the evolution of indicator sexual traits of antiparasitic capabilities with a genetic basis, and, thus, immunity would evolve rapidly due to sexual selection (Schmid-Hempel 2011). This theoretical background, however, does not consider the influence of one of the two lines of antiparasitic defences, tolerance to parasitism.

Defensive resistance traits are characteristics that prevent or release hosts from parasitism. Instead, defensive tolerance applies to host characteristics that reduce the negative fitness effects of parasitism without affecting parasite fitness (Råberg et al. 2007). Immune responses are examples of resistance defences because they eliminate parasites from hosts, while red blood cell disorders that reduce the incidence of malarial parasites without affecting parasite loads are considered defensive tolerance (Råberg et al. 2009). Defensive tolerance of parasites is actually considered just as important as resistance (Svensson and Råberg 2010; Medzhitov et al. 2012; Medina and Langmore 2016). Although plant biologists recognized the importance of defensive tolerance (i.e. acceptance of parasitism while minimizing the harm caused by the parasite) in antagonistic interactions more than one century ago, this has been neglected until recently by animal biologists, who have usually concentrated on the study of resistance (Råberg et al. 2009; Sorci 2013; Råberg 2014). Indeed, tolerance has been largely ignored in studies of antagonistic interactions in animals, and a good example is that the theory explaining parasite-mediated sexual selection does not consider tolerance.

Both resistance and tolerance are likely to be costly and may have equivalent short-term benefits for individual hosts (Roy and Kirchner 2000). They can be considered complementary defensive strategies, and, consequently, the evolution of one may be related to the evolution of the other (Råberg et al. 2007; Ayres and Schneider 2008; Sorci 2013; Willink and Svensson 2017). When focusing on defensive resistance, animal biologists usually use the term “tolerance” to indicate the absence of defensive resistance (Moskat et al. 2009; Martínez and Merino 2011; Anaya-Rojas et al. 2016), therefore, assuming that resistance and tolerance are two extremes of the same process. However, from an ecological and evolutionary perspective, defensive resistance and defensive tolerance are mutually different. Host resistance will negatively affect parasite fitness and, thus, reduce parasite population size. Instead, defensive tolerance of hosts will not affect parasite fitness, and, thus, the population of parasites will increase with time (i.e., higher prevalence of infection in the host population (Miller et al. 2006; Boots 2008)). Moreover, tolerance will select for few or no counter-adaptations in parasites (but see Little et al. 2010), but will result in non-virulent parasitism (Little et al. 2010; Sternberg et al. 2013). Consequently, tolerance will strongly affect coevolutionary dynamics between parasites and their hosts. Evolutionary theory predicts that the spread of resistance genes will select for new infectious parasites able to infect the common hosts resistant to other parasite phenotypes. This will result in negative frequency-dependence with limit cycles and maintenance of polymorphisms in parasites and hosts (Dybdahl and Lively 1998; Nuismer et al. 2005). In contrast, tolerance genes can spread to fixation while allowing higher parasite prevalence (Roy & Kirchner 2000). Consequently, the degree of variation in both resistance and tolerance is expected to influence host-parasite dynamics (Hayward et al. 2014), including the evolution and diversification of the different players (Best et al. 2014).

As proposed for defensive resistance, defensive tolerance of individual hosts against more prevalent parasites in the population could have been favoured by sexual selection. Thus, similar to what Hamilton and Zuk (1982) proposed for secondary sexual traits indicating defensive resistance, females selecting males with traits reflecting tolerance capability would gain tolerance genes for their offspring. Thus, parasite-mediated sexual selection may also favour the evolution of sexually selected indicators of defensive tolerance. The underlying mechanisms would be similar to those described for characters reflecting defensive resistance (see, Andersson 1994). The only difference is that in this case, negative effects of parasites should not directly alter male showiness, but indirectly because hosts with better defensive tolerance would have larger energy budgets that sexually selected traits would reflect.

Our main aim here is to propose that defensive tolerance can be favoured by sexual selection. We exemplify this possibility by analysing the expected relationship between known sexually selected traits of barn swallows (*Hirundo rustica*) and tolerance of parasites in a population studied for more than 30 years. Tolerance is usually estimated as the reaction norm of fitness related variables in relation to parasite load; the more negative the slope the lower the tolerance (Råberg et al. 2009). If tolerance to parasitism was favoured by sexual selection, we should expect a positive association between level of tolerance and expression of sexually selected characters. Here we used information on two sexually selected traits in barn swallows; tail length (Møller 1994b) and nest size (Soler et al. 1998a; Vergara et al. 2012), and loads of hematophagous mites (*Ornithonyssus bursa*), feather mites, louse flies, and chewing lice. Complete information was unavailable for all parasites and sexually selected characters, and, thus, sample sizes differed among statistical tests. Moreover, since reliability of tolerance estimates would depend on number of years used to estimate the slope of the association between reproductive success and parasitism, estimates were weighted by sample size in the analyses. We explore the expected negative association between parasitism and reproductive success, and between sexually selected traits and parasite loads, which will allow a more complete assessment of the importance of parasite-mediated sexual selection, and sexual selection mediating the evolution of host defences (i.e. resistance and tolerance of parasites).

## Material and methods

### Study population

APM studied barn swallows at Kraghede (57º12’N, 10º00’E), Denmark in an open farmland habitat during 1971–2016, with individual-based data being recorded since 1984. This study area of 15 to 45 km^2^ (the latter since 1988) consists of scattered farms and houses interspersed by fields and meadows where the main crops are wheat and potatoes. Hedgerows, small plantations, ponds and ditches are dispersed across the study area. Barn swallows breed inside barns and houses and, rarely, under bridges.

### General field procedures

APM captured barn swallows weekly during April-August 1984–2016 by using mist nets placed in front of doors and windows of farm buildings (Møller 1994b). All individuals were provided with an individually numbered aluminium ring and a colour ring for individual identification. Upon capture APM measured length of the two outermost tail feathers, and length of wings and central tail feathers with a ruler to the nearest mm (Møller 1994b). In addition, a number of other morphological characters were measured with a digital calliper to the nearest 0.01 mm, and body mass with a Pesola spring balance to the nearest 0.1 g. A small feather sample was collected from the throat, back and breast. Parasites were quantified as described below. Subsequently all individuals were released at the site of capture.

Adult barn swallows were assigned to nests using binoculars, allowing for assessment of reproductive success. The contents of nests were checked weekly. Breeding success was determined as the number of nestlings at fledging based on the number of nestlings at the last nest visit (i.e. breeding success). All nests were checked for the presence of a second clutch and yearly breeding success was estimated as the addition of that recorded for the first and second reproductive attempt (the latter only when a second clutch was laid).

### Parasites

APM quantified the abundance of ectoparasites in captured adult barn swallows as described in detail elsewhere (Møller 1991a, 1994b). The abundance of the hematophagous mite *Ornithonyssus bursa* was quantified as the number of individuals recorded on adults while handling the birds when recording measurements as described above. The abundance of the chewing louse *Brueelia* sp. was recorded as the number of holes produced in the wing and tail feathers since this estimate is strongly positively correlated with the abundance of lice observed during handling (Møller 1991a). This estimate was also highly repeatable during subsequent recaptures during the same and during following years (Møller 1991a, 1994a). The abundance of feather mites in the wing and tail feathers was recorded from the number of individuals seen when the feathers were held against the light. This measure of abundance of feather mites was highly repeatable when assessed on subsequent capture events the same season, but also in subsequent seasons (Møller 1991a, 1994b). The abundance of louse flies of the species *Ornithomyia biloba* was quantified as the number of adults seen while recording phenotypic characters as described above. The abundance of chewing lice was significantly heritable (Møller et al. 2004) as was the abundance of hematophagous *Ornithonyssus bursa* (Møller 2000). The associations between parasitism and sexually selected traits and viability of swallows are shown in Table 1.

**Table 1:** Known relationship between parasitism by hematophagous mites *Ornithonyssus bursa*, chewing lice (*Brueelia sp.*), feather mites and louseflies (*Ornithomyia biloba*) and tail length, and fitness related variables (reproductive success and survival)

Parasite	Related to fitness?	Sign of the relationship with tail length	Reference
Hematophagous mite	Yes	(−)	(Møller 1991a)
Chewing louse	Yes	(−)	(Møller 1991a)
Feather mites	No	(−)	(Møller 1991a)
Louse flies	Yes		(Saino et al. 1998)

### Sexually selected traits

Romano et al. (2017) provide a general overview of sexual selection in barn swallows. APM recorded the two sexually selected traits as follows. Tail length was measured when an individual was captured the first time any given year. Tail length affects mating success and other components of sexual selection as shown by observations and experiments (Møller 1988, 1990b, 1991b, 1992). It is highly repeatable (Møller 1991b, 1994b), significantly heritable (Møller 1991b; Saino et al. 2003) and correlated with the abundance of parasites and immunity (Møller 1990a, 1991a).

Nest size is measured as a fraction of a spheroid, which can be approximated as the product of length, width and height of the nest (for details, see Møller 2006). Nest size involves sexual selection because females whose males build larger nests invest more in parental care (Soler et al. 1998a). It is heritable and is phenotypically and genetically correlated with tail length in males (Møller 2006). Nest size in the barn swallow is also correlated with immunity (Soler et al. 2007).

### Statistical approach

Abundance of chewing lice and feather lice, as well as nest size was log_10_-transformed before analyses to achieve normal distributions. Prevalence of the hematophagous mite *Ornithonyssus bursa* and louseflies was relatively low and thus we classified these variables as binomially distributed in our analyses. Reproductive success and tail length were already normally distributed. ESM-Appendix 1 shows the number of individuals captured per study year for which we collected information on reproductive success, sexually selected characters and parasitism.

Parasitism and reproductive success appeared negatively related only for hematophagous mites and louse flies (see Results), and, thus, we only used these two parasites to estimate tolerance and to explore the expected associations with secondary sexual traits. Moreover, only three of the 1732 samples with information of nest size were parasitized by hematophagous mites, which prevents us to explore the associations with parasitism.

Tolerance was estimated as the slope of the relationship between reproductive success (dependent variable) (i.e. annual fledging production) and parasitism (independent factor) (Råberg et al. 2009; Sorci 2013; Medina and Langmore 2016) experienced by individual swallows followed for at least three reproductive seasons and that suffered from parasitism for at least one reproductive season. Tolerance to hematophagous mites was estimated with information on individuals that were captured in 3 (*n = *30), 4 (*n = *8), 5 (*n = *6) and 6 (*n = *3) different years. For all of these individuals we had information on tail length, but no information on nest size was available. Tolerance to chewing lice was estimated with information on swallows that were captured 3 (*n = *172), 4 (*n = *61), 5 (*n = *24), 6 (*n = *7) and 7 (*n = *3) different years. For all of these individuals we had information on tail length. Information on nest size were available for 97, 35, 9, 2, and 1 individuals for which we had information on parasitism and reproductive success in 3, 4, 5, 6 and 7 different years respectively. Thus, we obtained a single tolerance value for individual and parasites, and explore the expected positive association with secondary sexual characters, for which we used mean values of measures performed in each of the study years that were used to estimate tolerance.

The expected negative association between fledgling production (dependent variable) and parasitism (independent factor) were explored in General Linear Mixed Models (GLMM) after statistically controlling for sex (fixed factor) and study year (random factor). The expected negative relationship between secondary sexual characters (dependent factor) and parasitism (independent factor) was explored separately for males and females in GLMM after controlling for the random effect of study year. Similarly, the expected positive associations between sexually selected traits and tolerance were tested for males and females separately General Linear Models (GLM). In this case, the analyses were weighted by sample size (i.e. number of study years) used to estimate tolerance because reliability of the estimated slopes depends on the number of years each individual was sampled.

In the case of binomially distributed parasites, we reported least-square means and standard error (SE) of dependent variables for parasitized and unparasitized individuals. For parasites with continuous information, we reported beta and SE of the association between dependent variables and parasite load or tolerance. All analyses were performed with Statistica 10.0 (Dell-Inc 2015).

## Results

### Parasitism and reproductive success

The expected negative association between reproductive success and parasitism was detected for hematophagous mites and chewing lice (Table 2). Moreover, sexual differences in the effect of parasitism on reproductive success were detected for chewing lice (Table 2). Since negative effects of parasites were only detected for hematophagous mites and chewing lice, for exploring the predicted associations between secondary sexual characters and parasitism (Hamilton and Zuk hypothesis), and tolerance to parasitism, we only considered parasitism by this group of parasites.

**Table 2:** Relationship between parasitism (independent factor) and number of fledglings produced per breeding season (dependent factor, i.e. reproductive success), after controlling for the effect of sex and the random effect of study year

Parasitized		Non-parasitized		Parasitism			Sex			Year			Parasitism * Sex		
*N*	mean (SE)	*N*	mean (SE)	*F*	df	*P*	*F*	df	*P*	*F*	df	*P*	*F*	df	*P*
Hematophagous mites															
479	6.08 (0.15)	3859	6.48 (0.05)	**6.01**	**1,4303**	**0.014**	0.91	1,4303	0.34	**5.69**	**32,4303**	** < 0.001**	0.30	1,4302	0.58
Louseflies															
428	6.04 (0.16)	4164	6.09 (0.05)	0.08	1,4557	0.771	3.82	1,4557	0.051	**4.97**	**32,4557**	** < 0.001**	0.18	1,4556	0.669
Chewing lice															
Beta	SE														
− 0.045	0.015			8.89	**1,4402**	**0.002**	**3.57**	**1,4402**	**0.059**	**5.10**	**31,4402**	** < 0.001**	**2.98**	**1,4401**	**0.084**
Feather mites															
− 0.008	0.017			0.19	1,3660	0.662	1.40	1, 3660	0.237	4.89	25,3660	< 0.001	0.02	1,3659	0.896

Because information on parasitism by different parasites was unavailable for the same individuals and study years, we ran separate models for different categories of parasites. Because of the relatively low prevalence of parasitism by hematophagous mites *Ornithonyssus bursa* or louse flies, we considered these to be binomially distributed variables. We report sample sizes and mean reproductive success for parasitized and non-parasitized individual after controlling for annual variation and sex (i.e. least square means). For parasitism by chewing lice and feather mites, we used log10-transformed values, which approach a Gaussian distribution, and we report beta (SE) values for the relationship between parasitism and reproductive success. The effects of the interaction between parasitism and sex were explored in separate models. Values in bold are those with associated p-values smaller than 0.1

### Parasitism and secondary sexually selected traits

After statistically controlling for the random effects of study year, the predicted negative association between parasitism and secondary sexual traits was not detected for hematophagous mites, but tail length of males and females were negatively related to chewing lice (Table 3). Thus, sexual selection favouring the exaggeration of tail length also favoured the evolution of resistance.

**Table 3:** Relationship between parasitism (independent factor) and nest size and tail length of male and female swallows (dependent factors), after controlling for the effects of study year (random factor)

	Parasitized		Non-parasitized		Parasitism			Study year		
	*N*	mean (SE)	*N*	mean (SE)	*F*	df	*P*	*F*	df	*P*
Hematophagous mites										
Tail length										
Males	234	107.9 (0.64)	1919	108.9 (0.2)	1.83	1,2119	0.176	**2.53**	**32,2119**	** < 0.001**
Females	245	91.27 (0.44)	1945	90.87 (0.16)	0.69	1,2156	0.406	**2.15**	**32,2156**	** < 0.001**
Chewing lice										
	**Beta**	**SE**								
Tail length										
Males	**− 0.075**	**0.019**			**15.77**	**1,2806**	** < 0.001**	**3.25**	**28,2806**	** < 0.001**
Females	**− 0.051**	**0.019**			**7.35**	**1,2872**	**0.007**	**2.76**	**31,2872**	** < 0.001**
Nest size										
Males	0.011	0.029			0.13	1,876	0.714	**33.91**	**12,876**	** < 0.001**
Females	**− **0.014	0.028			0.24	1,896	0.624	**35.15**	**12,896**	** < 0.001**

Because information on parasitism by different parasites was unavailable for the same individuals and study years, we ran separate models for different categories of parasites. Because of the relatively low prevalence of parasitism by the hematophagous mite *Ornithonussus bursa* we considered this to be binomially distributed. No data were available to test the association between hematophagous mites and nest size. We report sample size and mean values of tail length of parasitized and non-parasitized individuals after controlling for annual variation (i.e. least square means). For parasitism by chewing lice we used log_10_-transformed values, which approach a Gaussian distribution, and we reported beta (SE) values for the relationship between parasitism and nest size and tail length of males and females. Values in bold are those with associated p-values smaller than 0.05

### Tolerance to parasitism and secondary sexually selected traits

**Fig. 1:**
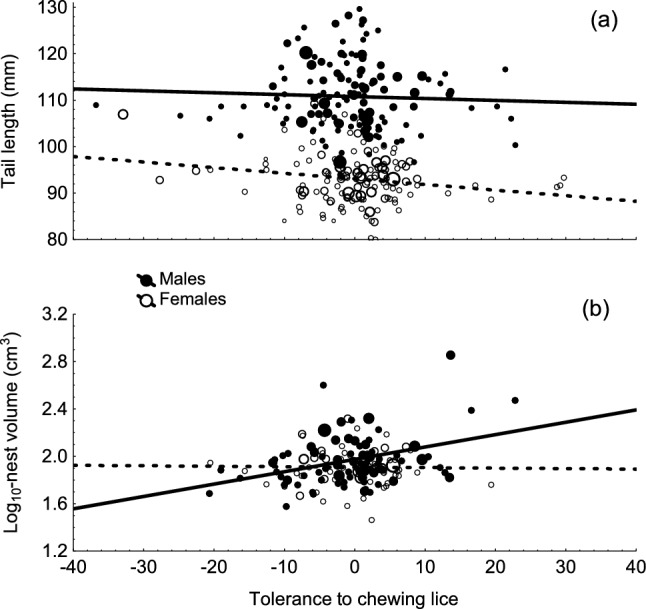
Relationship between tolerance to chewing lice and tail length **A** and log_10_ –transformed volume of nest material **B**. Lines are regression lines shown separately for males (solid) and females (dotted lines). Size of points is proportional to the number of years with available information used to estimate tolerance to chewing lice

The slopes of the association between reproductive success and parasitism by hematophagous mites (weighted mea*n = *− 0.25, CI = − 0.80–0.29) and by chewing lice (weighted mea*n = *− 1.11; CI = − 2.43–0.22) were on average negative. Tolerance to hematophagous mites was not associated with tail length of females or males (Table 4). Tolerance of male swallows to chewing lice was positively associated to nests size, but not tail length (Table 4, Fig. 1). In the case of females, nest size was not associated with tolerance to chewing lice, but tail length was negatively related to tolerance (Table 4, Fig. 1). Thus, sexual section favouring the exaggeration of nest size would also favour the evolution of tolerance.

**Table 4:** Relationship between tolerance to parasitism (independent factor) and nest size and tail length of male and female swallows (i.e. average values of years used for estimating tolerance for each individual) (dependent factor)

	Beta	SE	*F*	df	*P*
Hematophagous mites					
Tail length					
Males	− 0.027	0.258	0.01	1,15	0.919
Females	0.159	0.187	0.73	1,28	0.400
Chewing lice					
Tail length					
Males	− 0.044	0.087	0.26	1,133	0.611
Females	− **0.205**	**0.087**	**5.50**	**1,126**	**0.021**
Nest size					
Males	**0.359**	**0.109**	**10.91**	**1,74**	**0.001**
Females	− 0.016	0.124	0.02	1,65	0.896

Tolerance was estimated as the slope of the relationship between breeding success (i.e. annual production of fledglings) and parasitism load estimated for the same individual in more than two years. Because information on parasitism by different parasites was unavailable for the same individuals and study years, we ran separate models for different groups of parasites. No data were available to test the association between tolerance to hematophagous mites and nest size. We report beta (SE) values of the predicted associations between tolerance and secondary sexual traits. Values in bold are those with associated p-values smaller than 0.05

## Discussion

Parasite mediated sexual selection has been the topic of intense research and enthusiastic debate for more than three decades. The subject has been reviewed several times (e.g. Clayton 1991; Møller and Saino 1994; Hamilton and Poulin 1997), and is extensively covered by most popular text-books on evolution (e.g. Futuyma 2005) and sexual selection (e.g. Andersson 1994). However, although defensive tolerance is currently recognized as an important antiparasitic line of defence, all theoretical background and empirical studies dealing with the role of sexual selection driving the evolution of antiparasite defences deal only with defensive resistance. Here, we suggest that this theory may also apply to defensive tolerance explaining the evolution of secondary sexual traits as indicators of defensive capability in general, i.e., including the possibility that secondary sexual traits signal defensive tolerance capabilities.

To exemplify this possibility we used a large data set of a Danish population of swallows, and estimated tolerance to hematophagous mites and to chewing lice. As sexually selected traits, we used tail length and nest size. A large amount of evidence supports that tail length is a secondary sexual trait of males but, apparently, not of females (Møller 1994b; Cuervo et al. 1996), while only correlative evidence suggests that nest size is a post-mating sexually selected trait in this species (Soler et al. 1998a). Our main findings are that nest size is positively related to tolerance to chewing lice of barn swallow males, but not to that of females, and that tail length of females and males was negatively associated with load of chewing lice. These results are in accordance with the possibility that tail length and nest size signal the ability of swallow males to, respectively, resist and tolerate parasitism by chewing lice. Bellow we first discuss the strength of this conclusion in swallows. Later, we discuss the importance of considering defensive tolerance for understanding the role of parasite-mediated sexual selection.

### Secondary sexual traits reflecting defensive tolerance and resistance in swallows

Among other factors, the strength of the detected associations depends on sample size, which in our data set varied depending on the specific parasites and secondary sexual traits of swallows. In comparison to parasitism by chewing lice, information on parasitism by hematophagous mites was available for a reduced number of swallows (see Appendix 1). Moreover, prevalence of hematophagous mites was quite low, which resulted in reduced sample size of parasitized individuals. Furthermore, sample sizes were drastically reduced when considering tolerance to hematophagous mites in our analyses (see df in Table 4). Thus, results dealing with parasitism by hematophagous mites, mainly those related to tolerance, should be considered cautiously.

Another possible problem is related to the reliability of the used estimates of tolerance. For instance, it can be argued that three points is not the optimal sample size to estimate the reaction norms between reproductive success and parasitism. Trying to solve this problem, our analyses were weighted by sample size used to estimate reaction norms.

Ideally, reliable measures of tolerance require a homogeneous environment, preferably a laboratory, and experimental variation in infection intensities (Råberg et al. 2009). However, this is not possible for natural populations because natural conditions are temporally and spatially variable and experimental parasitism is ethically problematic. Rather, we used information from different breeding seasons, which implies environmental variation that might affect infection intensities, fitness, and, thus, tolerance (Tiffin and Inouye 2000). For instance, environmental conditions could influence both availability of ectoparasites in the environment and reproduction success of hosts in the same direction (i.e., years of high availability of resource for hosts and for ectoparasites). If that was the case, positive values of tolerance would be predicted, which at a first glance may appear counterintuitive (see Råberg et al. 2009). Since parasite loads of tolerant individuals, but not those of resistant individuals, would more closely reflect availability of parasites in the environment, reproductive success and parasitism should be positively related. Thus, even considering possible problems of estimating tolerance in variable environments and with natural parasitism, the approach we followed (i.e., considering different reproductive events) should be considered appropriate for exploring the hypothesis tested. In any case, it is worth to mention here that exploring predictions in our swallow population serves to exemplify the importance of considering tolerance in parasite-mediate sexual selection, which was our principal aim.

Tail length of barn swallows showed a negative association with chewing lice parasitism in accordance with the traditional view of the Hamilton and Zuk hypothesis. This relationship was not detected for nest size, which has a recognized sexual component in birds (Soler et al. 1998a, 1998b), but males with large nests showed high levels of defensive tolerance to chewing lice. Traits related to defensive tolerance likely have a significant genetic component (Roy and Kirchner 2000), and nest size is a heritable character in barn swallows (Møller 2006). Thus, because females invest differentially in reproduction when mated with males building exaggerated nests (Soler et al. 1998a), sexual selection would affect the evolution of defensive tolerance, while selection due to parasitism mediates the evolution of nest size.

Defensive resistance and tolerance may be considered complementary antiparasite strategies (Råberg et al. 2007; Ayres and Schneider 2008; Willink and Svensson 2017). Female barn swallows, by selecting mates with exaggerated tail length, or investing differentially in reproduction when paired with those that built large nests, will select for good genes for their offspring related to parasite resistance or tolerance, respectively. Characters reflecting resistance and tolerance to parasitism can be genetically linked (Stowe 1998) as it appears to be the case in swallows for which tail length of males and nest size are negatively related (Soler et al. 1998a). Genetic linkage between resistance and tolerance is important because frequency-dependent selection on resistance will inhibit the fixation of both resistance and tolerance (Roy and Kirchner 2000). Moreover, the expected advantage of resistance and tolerance likely depends on environmental conditions including parasite virulence (Sorci 2013). Thus, we speculate that depending on environmental conditions, females may select mates highly resistant or highly tolerant to parasitism, which would facilitate maintenance of genetic variation in defensive resistance and defensive tolerance. Thus, considering defensive tolerance in scenarios of parasite-mediated sexual selection may also contribute to understanding how genetic variation in antiparasite defence is maintained in natural population; the so-called lek paradox (see Andersson 1994).

### The importance of considering defensive tolerance in scenarios of parasite-mediated sexual selection

The debate about the Hamilton and Zuk hypothesis mainly derives from inconsistent results when confronting assumptions and predictions of the hypothesis in empirical studies. Most results from experimental studies were consistent with general assumptions of parasite-mediate sexual selection, but not correlational findings, which were mainly derived from natural populations (Clayton 1991). The association between parasitism and the expression of secondary sexual traits is perhaps the conundrum prediction of the hypothesis that a relatively high number of published results do not fulfil. At the intraspecific level, Hamilton and Poulin (1997) evaluated published papers testing the expected negative association between parasitism and expression of secondary sexual traits, concluding that “As a whole intraspecific correlations between parasite load and male showiness provided very little support for the hypothesis”. More recent non-experimental results do not change this view since they report either negative (Brawner et al. 2000; Del Cerro et al. 2010; Vergara et al. 2012; Molnár et al. 2013), no (Borgia 1986; Borgia and Collis 1990), or even positive relationships (Buczek et al. 2016; Trigo and Mota 2016) between sexual traits and parasitism. At the interspecific level, the hypothesis predicts that sexual ornamentation should be more exaggerated in species with many parasites (Andersson 1994). Such a relationship was found among subfamilies of birds (Read 1988) and in many other studies of insects, fish, amphibian, reptiles, birds and mammals (Schmid-Hempel 2011). However, most of these associations were likely confounded by interspecific covariation of parasitism, secondary sexual traits, and phylogenetic similarities. In fact, after controlling for common ancestry and other sources of errors, secondary sexual traits and prevalence of malaria-like parasites resulted in no or weakly positive correlations (Read and Weary 1990; Garamszegi and Møller 2012). Thus, currently, the overall evidence in favour of the Hamilton and Zuk hypothesis is mixed.

In our opinion, this weak support may be due to the fact that neither hypothesis nor predictions considered tolerance as an antiparasite defence signalled by sexually selected traits. It is fully feasible that particular sexually selected characters showed defensive tolerance of hosts to particular parasites. If we imagine a group of hosts experiencing exactly the same environmental conditions and parasite burden, those with a higher level of defensive tolerance would also have more resources available for sexual display. Thus, individuals with higher level of defensive tolerance will be those displaying more exaggerated sexual characters. If that was the case, a negative relationship between parasite load and the expression of a sexually selected character would no longer be a prediction of parasite defence mediated sexual selection. It is still possible that sexual attraction relates to tolerance capability of individuals if the sexually selected traits were related to the slope of the relationship between reproductive success and parasite burden, which is considered an appropriate estimation of tolerance. Thus, by extending the Hamilton and Zuk hypothesis to consider both defensive resistance and defensive tolerance as heritable antiparasite defences, failing to detect a negative relationship between parasitism and secondary sexual traits does not invalidate the hypothesis, but open the possibility that defensive tolerance and secondary sexual traits are related to each other.

To summarize, our results from analyses of data from a population of individually marked barn swallows studied during the last 30 years served to exemplify the importance of considering antiparasite defensive tolerance in order to understand scenarios of parasite-mediated sexual selection on secondary sexual characters that indicate heritable antiparasite defence capabilities. Extending what Hamilton and Zuk proposed for sexual traits reflecting defensive resistance, some of these traits may also reflect defensive tolerance. This extension of the hypothesis may explain the mixed results when testing the main predictions and help to explain how genetic variation is maintained in association with temporal variation in environmental conditions. We consider that this contribution may prompt scientists to have another look at the theory of parasite-mediated sexual selection in a wider context that not only includes defensive resistance, but also promotes further research considering the role of defensive tolerance.

### Supplementary Information

Below is the link to the electronic supplementary material.

Supplementary file1 (DOCX 16 KB)

## Data Availability

The datasets used and/or analysed during the current study are available from the corresponding author on reasonable request.
