# Supplementary material for: Defensive tolerance to parasitism is correlated with sexual selection in swallows
Source: Oecologia. 2023 Jul 18;203(3-4):267–76. doi: 10.1007/s00442-023-05419-5 (PMC10684419; doi:10.1007/s00442-023-05419-5)
Supplement: Supplementary file 1 — Supplementary file1 (DOCX 16 KB) [file 442_2023_5419_MOESM1_ESM.docx]

ESM-Appendix 1. Number of males and females captured per study year with indication of number of individuals with information of reproductive success (R. success), sexually selected traits (Tail length and nest size) and parasitism by hematophagous (Haem.) mites, Chewing (Chew.) lice, feather mites and louse flies.

|  | Individual captured | | | R. success | Sexually selected traits |  | Parasitism | | | |
| --- | --- | --- | --- | --- | --- | --- | --- | --- | --- | --- |
| Year | ♂♂ | ♀♀ | TOT | Fledgl. prod. | Tail length | Nest size | Haem. mites | Chew. lice | Feather mites | Louse flies |
| 1984 | 25 | 23 | 48 | 48 | 48 | 0 | 46 | 0 | 0 | 48 |
| 1985 | 20 | 15 | 35 | 35 | 35 | 0 | 31 | 6 | 0 | 35 |
| 1986 | 29 | 25 | 54 | 54 | 54 | 0 | 50 | 25 | 0 | 54 |
| 1987 | 46 | 43 | 89 | 88 | 89 | 0 | 86 | 42 | 0 | 89 |
| 1988 | 138 | 144 | 282 | 172 | 282 | 0 | 161 | 282 | 0 | 282 |
| 1989 | 141 | 152 | 293 | 179 | 293 | 0 | 165 | 293 | 7 | 293 |
| 1990 | 133 | 153 | 286 | 169 | 285 | 0 | 161 | 286 | 0 | 286 |
| 1991 | 147 | 145 | 292 | 158 | 290 | 0 | 154 | 291 | 0 | 292 |
| 1992 | 130 | 128 | 258 | 218 | 255 | 0 | 188 | 258 | 256 | 258 |
| 1993 | 73 | 73 | 146 | 136 | 146 | 0 | 129 | 146 | 146 | 146 |
| 1994 | 52 | 52 | 104 | 84 | 104 | 0 | 80 | 104 | 104 | 104 |
| 1995 | 29 | 35 | 64 | 49 | 62 | 0 | 47 | 62 | 62 | 64 |
| 1996 | 57 | 58 | 115 | 99 | 113 | 0 | 91 | 113 | 113 | 115 |
| 1997 | 103 | 92 | 195 | 159 | 195 | 0 | 151 | 195 | 195 | 195 |
| 1998 | 92 | 91 | 183 | 154 | 182 | 0 | 150 | 183 | 183 | 183 |
| 1999 | 63 | 61 | 124 | 97 | 123 | 0 | 96 | 124 | 124 | 124 |
| 2000 | 80 | 99 | 179 | 166 | 177 | 0 | 133 | 179 | 179 | 179 |
| 2001 | 80 | 77 | 157 | 141 | 154 | 0 | 127 | 157 | 157 | 157 |
| 2002 | 93 | 91 | 184 | 128 | 184 | 0 | 118 | 184 | 184 | 184 |
| 2003 | 120 | 120 | 240 | 185 | 235 | 0 | 170 | 240 | 238 | 240 |
| 2004 | 154 | 164 | 318 | 200 | 317 | 160 | 202 | 318 | 318 | 318 |
| 2005 | 71 | 75 | 146 | 116 | 146 | 122 | 114 | 146 | 146 | 146 |
| 2006 | 135 | 130 | 265 | 157 | 263 | 104 | 144 | 265 | 265 | 265 |
| 2007 | 133 | 132 | 265 | 187 | 264 | 168 | 187 | 265 | 265 | 265 |
| 2008 | 123 | 111 | 234 | 220 | 232 | 188 | 210 | 234 | 234 | 234 |
| 2009 | 141 | 139 | 280 | 257 | 277 | 233 | 237 | 280 | 280 | 280 |
| 2010 | 126 | 102 | 228 | 203 | 227 | 185 | 194 | 227 | 227 | 227 |
| 2011 | 109 | 104 | 213 | 182 | 208 | 160 | 191 | 213 | 213 | 213 |
| 2012 | 66 | 63 | 129 | 120 | 129 | 106 | 121 | 129 | 129 | 129 |
| 2013 | 79 | 84 | 163 | 124 | 161 | 111 | 128 | 163 | 163 | 163 |
| 2014 | 58 | 62 | 120 | 103 | 119 | 91 | 103 | 120 | 120 | 120 |
| 2015 | 57 | 71 | 128 | 111 | 126 | 95 | 111 | 128 | 127 | 128 |
| 2016 | 71 | 52 | 123 | 93 | 123 | 77 | 95 | 123 | 123 | 123 |
| Total | 2974 | 2966 | 5940 | 4592 | 5898 | 1800 | 4371 | 5781 | 4558 | 5939 |
